# Potential Synergistic Antibiotic Combinations against Fluoroquinolone-Resistant *Pseudomonas aeruginosa*

**DOI:** 10.3390/ph15020243

**Published:** 2022-02-18

**Authors:** Ashish Kothari, Neeraj Jain, Shyam Kishor Kumar, Ankur Kumar, Karanvir Kaushal, Satinder Kaur, Atul Pandey, Amit Gaurav, Balram Ji Omar

**Affiliations:** 1 Department of Microbiology, All India Institute of Medical Sciences Rishikesh, Rishikesh 249203, India; ashish.sci@aiimsrishikesh.edu.in (A.K.); ankurk591@gmail.com (A.K.); 2 Department of Medical Oncology, All India Institute of Medical Sciences Rishikesh, Rishikesh 249203, India; neeraj.monc@aiimsrishikesh.edu.in; 3 Division of Cancer Biology, Central Drug Research Institute, Lucknow 226031, India; 4 Department of Microbiology, All India Institute of Medical Sciences Deoghar, Deoghar 814152, India; dr.shyamkishor84@gmail.com; 5 Department of Biochemistry, All India Institute of Medical Sciences Rishikesh, Rishikesh 249203, India; karanvirkaushal@gmail.com; 6 Department of Microbiology, Postgraduate Institute of Medical Education and Research, Chandigarh 160012, India; satinder.silky20@gmail.com; 7 Department of Ecology, Evolution and Behavior, The Hebrew University of Jerusalem, Jerusalem 9190401, Israel; 8 Department of Ecology and Evolutionary Biology, University of Michigan, Ann Arbor, MI 48109, USA; 9 Satbiome, Dehradun 248007, India

**Keywords:** synergy, quinolones, polymyxins, PMQR, QRDR, resistant, *Pseudomonas aeruginosa*

## Abstract

The rise in multiple-drug-resistant (MDR) phenotypes in Gram-negative pathogens is a major public health crisis. *Pseudomonas aeruginosa* is one of the leading causes of nosocomial infections in clinics. Treatment options for *P. aeruginosa* have become increasingly difficult due tdo its remarkable capacity to resist multiple antibiotics. The presence of intrinsic resistance factors and the ability to quickly adapt to antibiotic monotherapy warrant us to look for alternative strategies like combinatorial antibiotic therapy. Here, we report the frequency of *P. aeruginosa* multidrug-resistant and extensively drug-resistance (XDR) phenotypes in a super-specialty tertiary care hospital in north India. Approximately 60 percent of all isolated *P. aeruginosa* strains displayed the MDR phenotype. We found highest antibiotic resistance frequency in the emergency department (EMR), as 20 percent of isolates were resistant to 15 antipseudomonal antibiotics. Presence of plasmids with quinolone-resistance determinants were major drivers for resistance against fluoroquinolone. Additionally, we explored the possible combinatorial therapeutic options with four antipseudomonal antibiotics—colistin, ciprofloxacin, tobramycin, and meropenem. We uncovered an association between different antibiotic interactions. Our data show that the combination of colistin and ciprofloxacin could be an effective combinatorial regimen to treat infections caused by MDR and XDR *P. aeruginosa*.

## 1. Introduction

The expanding frequency of multidrug-0resistant pathogens and the virtually dry pipeline of new antibiotics have created a formidable challenge for our public health settings [1,2]. Infections due to Gram-negative pathogens, especially *Pseudomonas aeruginosa*, are responsible for high mortality around the globe [3,4,5]. *P. aeruginosa* is responsible for blood infections, respiratory tract infections, skin infections, urinary tract infections, and surgical site infections [6,7,8]. The presence of high-level intrinsic and acquired resistance determinants in this pathogen often results in poor clinical outcomes. Frequent reports of multidrug-resistant (MDR) and extensively drug-resistant (XDR) *P. aeruginosa* isolates from hospitals in low- and middle-income countries make the situation even more problematic for public health departments [9,10,11,12].

Treatment options available for *P. aeruginosa* infections are limited and shrinking rapidly [13]. Four antibiotic classes, i.e., fluoroquinolones, β-lactams, aminoglycosides, and polymyxins, are routinely prescribed against *P. aeruginosa* infections [7,13]. Fluoroquinolones are among the most active antibiotics against *P. aeruginosa*. However, frequent chromosomal mutations and horizontally acquired resistance elements enable this bacterium a swift escape against most of these antibiotics. Different resistance determinants such as target site (DNA gyrase and DNA topoisomerase IV) modifications, alteration in membrane permeability, and active efflux are responsible for fluoroquinolones resistance [14,15]. Both chromosomal-mediated and plasmid-mediated resistance determinants have been reported in *P. aeruginosa* [16]. Fluoroquinolone resistance may arise due to spontaneous mutations in chromosomal DNA gyrase and topoisomerase IV genes, and these genomic regions are called quinolone-resistance-determining regions (QRDR) [17]. However, in the recent past, plasmid-mediated quinolone resistance (PMQR) has been increasingly reported in Gram-negative bacteria all over the world [18,19]. Multiple *qnr* determinants (*qnrA*, *qnrB*, *qnrS*, and *qnrD*) have been identified in Gram-negative pathogens that are responsible for quinolones resistance [20,21]. Additionally, some plasmid-based novel determinants such as a modified acetyltransferase gene (*aac(6′)-Ib-cr*) and the efflux pump gene (*qepA*) have also been characterized in the recent past [22,23].

The lack of discovery of new antibiotics, especially antibiotics targeting Gram-negative bacteria, further contributes to the difficulty and often necessitates using existing antibiotics in a more innovative way such as combinatorial therapy [24]. Combinatorial therapy is an attractive approach against MDR and XDR isolates of *P. aeruginosa* [25,26,27]. Combinatorial therapy can provide multifaceted benefits in clinics as it can increase the empirical coverage against other pathogens, suppress the emergence of resistant phenotypes, and decrease toxicity [25,28]. *P. aeruginosa* has an additional worrisome feature, as it can rapidly adapt against antimicrobial monotherapy, and thus combinatorial therapy can also alleviate this inherent problem [29,30,31].

Many antibiotic interactions of fluoroquinolones have been reported against different pathogens, including *P. aeruginosa*, in the past [32]. However, it is still unclear how these complex antibiotic interactions depend on the level of antibiotic resistance. To better understand the interactions among routinely prescribed antibiotics against *P. aeruginosa*, we carried out extensive screening of antibiotic interactions against XDR *P. aeruginosa*. Here, we describe the co-existence of QRDR and PMQR, complex interconnection with the type of PMQR, and antibiotic interaction. We also show the interspecies transferability and stability of PMQR. This study aims to identify the optimal combinatorial therapy approaches effective against XDR *P. aeruginosa*.

## 2. Results

### 2.1. A High Level of Antibiotic Resistance Is Observed among P. aeruginosa

**Figure 1:**
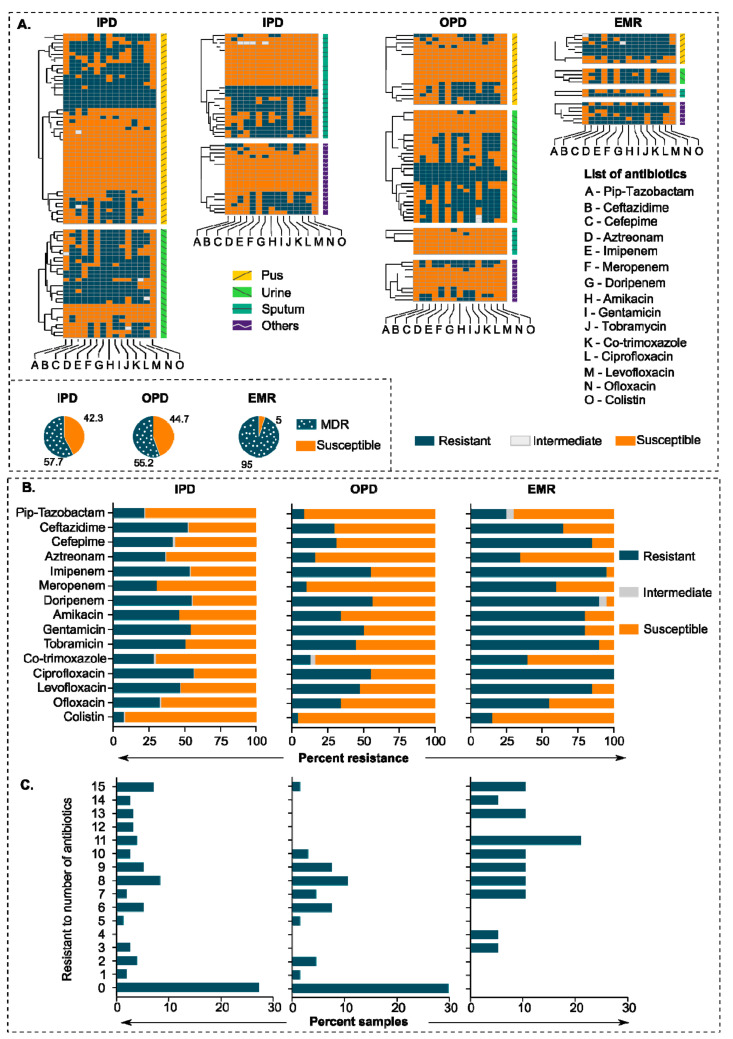
Antibiotic resistance pattern in clinical strains of *P. aeruginosa*. (**A**) Heatmap showing an antibiotic resistance pattern of 243 clinical strains of *P. aeruginosa* against 15 antipseudomonal antibiotics. IPD, OPD, and EMR represent Inpatient Department, Outpatient Department, and Emergency Department, respectively. The source of isolation is indicated on the left side of the heatmaps. (**B**) Percent resistance of all samples (clinical isolates) in different departments against 15 antipseudomonal antibiotics. (**C**) Bar graphs showing a frequency of the number of antibiotics against department-wise *P. aeruginosa* resistance percentage.

We collected 243 clinical strains of *P. aeruginosa* from various departments in a large tertiary care hospital located in northern India (All India Institute of Medical Sciences Rishikesh, Uttarakhand, India). About 60% of *P. aeruginosa* strains showed multiple drug-resistant phenotypes (resistant to three or more drug classes). We observed the highest rates of resistance for the fluoroquinolones, with resistance to ciprofloxacin, ranging from 50 to 100% in IPD (inpatients) and EMR (emergency) departments, respectively (Figure 1A,B). Surprisingly, we also found a high level of resistance against the last resort of antibiotics, i.e., carbapenems, with an average resistance of >50% in all departments. *P. aeruginosa* isolates from the EMR department showed higher resistance rates for fluoroquinolones, β-lactams, aminoglycosides, co-trimoxazole, and colistin than general trends for hospitalized patients. Overall, we observed the lowest resistance rates against ofloxacin, amikacin, and meropenem among fluoroquinolones, aminoglycosides, and carbapenems classes, respectively. Similarly, the resistance rate for colistin was lowest among all tested antipseudomonal antibiotics. About 10 and 20% of the *P. aeruginosa* isolates from the EMR department were resistant to 10 and 15 antibiotics, respectively (Figure 1C).

### 2.2. Antibiotic–Antibiotic Interaction Screening Results Showed Colistin–Ciprofloxacin Is the Most Effective Antibiotic Combination against MDR P. aeruginosa

**Figure 2:**
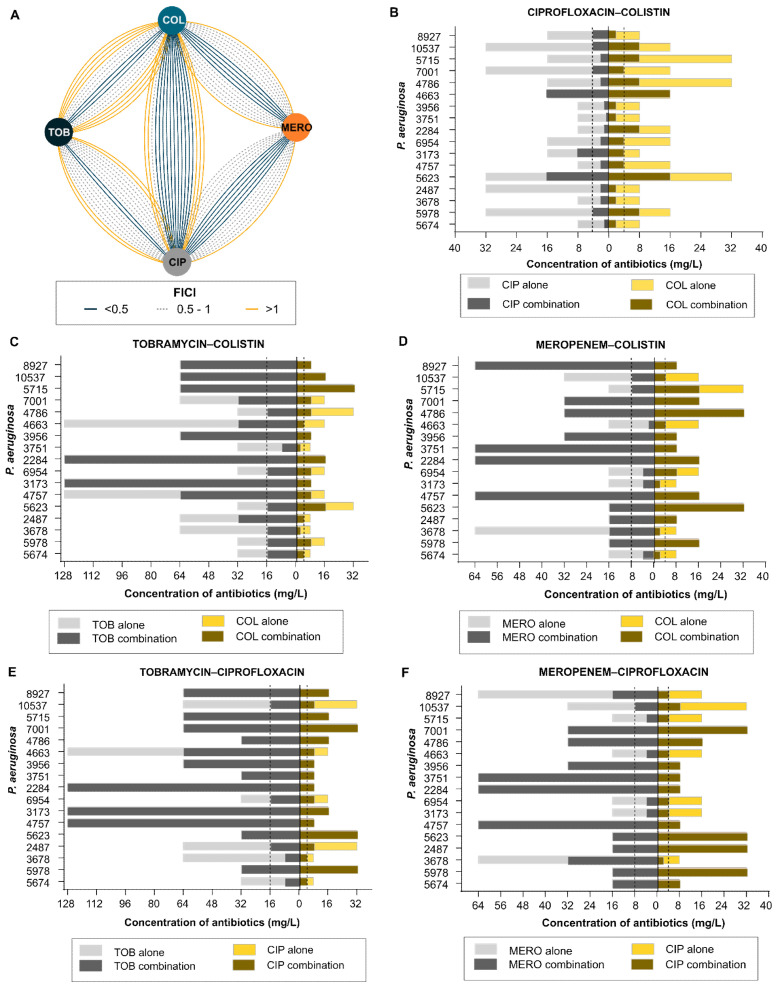
(**A**) Antibiotic–antibiotic interaction network of four antipseudomonal antibiotics (colistin, meropenem, ciprofloxacin, and tobramycin) in 17 extensive drug-resistant clinical strains of *P. aeruginosa*. Nodes represent different antibiotics, edges represent Fractional Inhibitory Concentration Index (FICI), i.e., synergy (FICI < 0.5; dark blue), partial synergy (FICI = 0.5–1; dashed gray), or no interaction (FICI > 1; yellow). Interaction network was created with Cytoscape version 3.8.0. (**B**) Minimum inhibitory concentration of ciprofloxacin–colistin; (**C**) tobramycin–colistin; (**D**) meropenem–colistin; (**E**) tobramycin–ciprofloxacin; (**F**) Meropenem–ciprofloxacin; alone (light gray and light yellow, respectively) or in combination (dark gray and dijon yellow, respectively) against 17 extensively drug-resistant clinical strains of *P. aeruginosa*. The dashed line represents the MIC breakpoint of respective antibiotics as defined by CLSI guidelines. The CLSI MIC breakpoint for *P. aeruginosa* resistance phenotype against ciprofloxacin, colistin, meropenem, and tobramycin is ≥4, ≥4, ≥8, and ≥16 mg/L, respectively [37].

To identify the most effective antibiotic combinations, we selected five antipseudomonal antibiotic pairs, i.e., colistin–ciprofloxacin, colistin–meropenem, colistin–tobramycin, ciprofloxacin–tobramycin, and ciprofloxacin–meropenem based on the previously reported regimens [33,34,35,36]. We tested them against 17 XDR *P. aeruginosa* strains. The combination of tobramycin with either colistin or ciprofloxacin showed the lowest rate of synergistic effect (only in three and two strains, respectively) (Figure 2A,C,E). Meropenem–colistin and meropenem–ciprofloxacin combinations showed synergy in five and six strains, respectively (Figure 2D,F). However, the ciprofloxacin–colistin combination showed synergy in 11 strains out of 17 strains. Additionally, in 9 out of 11 strains, the concentration of ciprofloxacin and colistin decreased below the respective breakpoint defined by CLSI guidelines (Figure 2B). The CLSI MIC breakpoint for *P. aeruginosa* resistance phenotype against ciprofloxacin, colistin, meropenem, and tobramycin is ≥4, ≥4, ≥8, and ≥16 mg/L, respectively [37].

### 2.3. Prevalence of Inter-Species Plasmid Transfer

**Figure 3:**
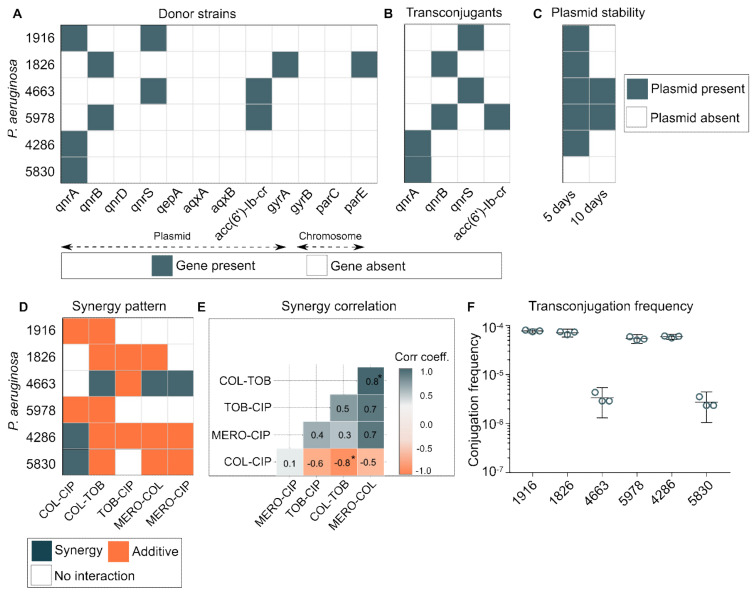
(**A**) Presence of quinolone resistance determinants in six *P. aeruginosa* strains. Twelve quinolone-resistant determinants were tested for their presence (*qnrA*, *qnrB*, *qnrD*, *qnrS*, *qepA*, *aqxA*, *aqxB*, *aac(6′)-Ib-cr*, *gyrA*, *gyrB*, *parC*, *parE*). (**B**) Four plasmid-mediated quinolone-resistant determinants genes (*qnrA*, *qnrB*, *qnrS*, and *aac(6′)-Ib-cr*) from clinical strains of *P. aeruginosa* were transferred to *E. coli* J53. (**C**) Inter-species plasmid stability in *E. coli* J53 after 5 and 10 days of transconjugation. (**D**) Antibiotic–antibiotic interaction pattern in six *P. aeruginosa* strains. (**E**) Antibiotic interaction correlation between five antibiotic interaction types displayed against six *P. aeruginosa* strains. The correlation matrix was computed using library (*ggcorrplot* version 0.1.3) in R. (**F**) Transconjugation frequency of six *P. aeruginosa* plasmids to *E. coli* J53. *: correlation significance value *p* < 0.05.

*P. aeruginosa* exploits multiple ways to tackle quinolone toxicity, as both plasmid-mediated and chromosomal-mediated resistance determinants play an important role. We tested twelve quinolone-resistant determinants for the presence of *qnrA*, *qnrB*, *qnrD*, *qnrS*, *qepA*, *aqxA*, *aqxB*, *aac(6′)-Ib-cr*, *gyrA*, *gyrB*, *parC*, and *parE* in 132 quinolone-resistant isolates of *P. aeruginosa*. The results are summarized in Supplementary Figure S1. Next, we selected six isolates based on the antibiotic resistance profile to check if plasmid-mediated quinolone-resistant (PMQR) determinants could be mobilized to other species through conjugation. We selected *E. coli* J53 Az^r^ as a recipient host strain. Our results show that quinolone resistance could be transferred by conjugation to all six PMQR-positive donors (Figure 3A). The frequencies of transconjugation varied between 10^−4^ to 10^−6^ cells per recipient cells for different donor strains. Four donor strains (1916, 1826, 5978, and 4286) transferred plasmid at a frequency of 10^−4^ cells per recipient cells and two remaining strains (4663 and 5830) at 10^−6^ (Figure 3F). Out of nine PMQR determinants, four genes (*qnrA*, *qnrB*, *qnrS*, and *aac(6′)-Ib-cr*) were successfully transferred to *E. coli* J53 (Figure 3B). Plasmids that had quinolone-resistance genes can provide a selective advantage to host under an environment with quinolone pressure; however, maintaining plasmids without any selective advantage (selection pressure) could be costly to the host cells. Hence, we checked the stability of these transferred plasmids without any selective advantage and found that five out of six transconjugants (1916, 1826, 5978, 4286, and 4663) retained the plasmid for five days, while two transconjugants (4663 and 5978) retained plasmid even for ten days without any selection pressure (Figure 3C). Next, we checked whether the type of antibiotic–antibiotic interactions (Figure 3D) have any correlation for a given strain. We calculated the Pearson correlation coefficient for these variables (i.e., FICIs against different antibiotic interactions) and found some intriguing associations; for example, the colistin–meropenem combination was positively correlated with the colistin–tobramycin combination (*r* = 0.8, *p* < 0.05), while colistin–ciprofloxacin and colistin–tobramycin were negatively correlated (*r* = −0.8, *p* < 0.05) (Figure 3E). Although statistically insignificant, meropenem–colistin was positively associated with meropenem–ciprofloxacin and tobramycin–ciprofloxacin combinations (*r* = 0.7) (Figure 3E). Comprehensive correlation analysis of antibiotic interactions for any bacterial pathogen could help prioritize an appropriate treatment regimen.

### 2.4. Transferred Plasmids Provide a High Level of Resistance

**Figure 4:**
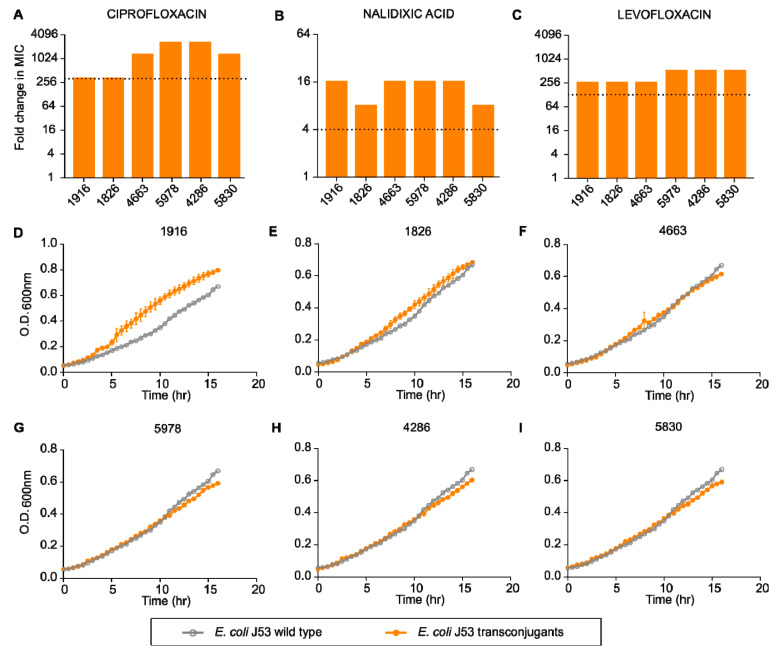
Fold change in minimum inhibitory concentration of different transconjugants compared to *E. coli* J53 against quinolones: (**A**) ciprofloxacin, (**B**) nalidixic acid, (**C**) levofloxacin. Relative growth of different transconjugants ((**D**) 1916, (**E**) 1826, (**F**) 4663, (**G**) 5978, (**H**) 4286, (**I**) 5830) compared to *E. coli* J53.

Plasmids are one of the major drivers of antibiotic resistance against multiple antibiotic classes. To test whether plasmid-mediated quinolone resistance determinants present in *P. aeruginosa* could be transferred to other closely related species, we performed a transconjugation experiment with *E. coli* J53 (an azide resistant recipient). We found that plasmids present in clinical strains of *P. aeruginosa* could be easily mobilized to *E. coli* and with a high frequency. Moreover, transferred plasmids could provide a high level of resistance to the recipient cells. Transconjugants displayed a 256- to 2048-fold increase in MIC against ciprofloxacin, 8- to 16-fold increase against nalidixic acid and 256- to 512-fold increase against levofloxacin (Figure 4A–C). This increase in MIC is much higher than the resistance breakpoints defined by CLSI guidelines for respective antibiotics [37]. Harboring external genetic material could result in reduced fitness for the host cells [38]. However, when we tested the growth pattern of transconjugants with the parental host strain, we did not find any significant reduction in the growth rate of transconjugants as compared to the parental strain (Figure 4D–I).

### 2.5. Quinolone Resistant Strains Show Reduced Drug Accumulation

**Figure 5:**
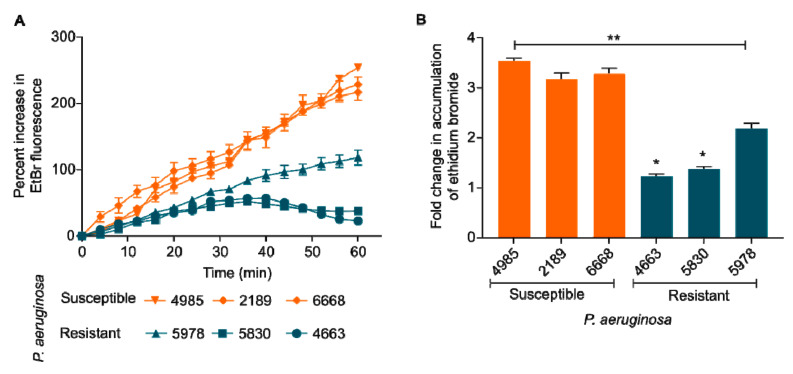
(**A**) Ethidium bromide (EtBr) accumulation kinetics. Quinolone-resistant *P. aeruginosa* (5978, 5830, and 4663) show reduced EtBr accumulation kinetics (i.e., higher efflux) as compared to quinolone-susceptible *P. aeruginosa* (4985, 2189, and 6668). (**B**) Fold change in the accumulation of EtBr after 60 min. One way ANOVA followed by Mann-Whitney test. *: *p* < 0.05. **: *p* < 0.01.

Bacterial efflux pumps play a central role in antibiotic resistance. Our results indicated that quinolone-resistant *P. aeruginosa* has higher efflux activity than the susceptible counterpart. Ethidium bromide is a common substrate for many efflux pumps in Gram-negative bacteria, and its reduced uptake inside these bacterial cells is indirectly proportional to the efflux activity [39,40]. We found that quinolone-resistant *P. aeruginosa* (5978, 5830, and 4663) show reduced EtBr accumulation kinetics (i.e., higher efflux) as compared to quinolone-susceptible *P. aeruginosa* (4985, 2189, and 6668) (Figure 5A). More specifically, the resistant strains showed 1.5 to 3 times decreased EtBr accumulation (*p* < 0.05) than the susceptible *P. aeruginosa* (Figure 5B).

## 3. Discussion

*P. aeruginosa* is currently one of the critical priority pathogens as defined by the World Health Organization [41]. They are also responsible for acute infections among patients admitted to ICUs. *P. aeruginosa* has been linked to more infection incidents and higher mortality rates in ICUs compared to other hospital wards. However, other clinical conditions of patients, such as pre-existing diseases, surgery, and immunosuppressive medications, play an important role in the mortality rate [6]. *P. aeruginosa* included in our study were mostly isolated from urine, followed by pus and sputum. Our data show a different trend as compared to previously reported clinical sources of isolated strains [42,43]. Our antibiotic resistance data show a high prevalence of MDR and XDR phenotypes in ICU patients as compared to IPD and OPD, which is also different from previously reported resistance frequencies among ICUs patients. We found a high prevalence of resistance against most of the antipseudomonal antibiotics except colistin and co-trimoxazole, which is in agreement with previously published surveys from different geographical locations [9,10]. However, we did not find any correlation between antibiotic resistance and sources of bacterial isolates, i.e., body site. For the treatment of *P. aeruginosa* infections at OPD departments in the All India Institute of Medical Sciences Rishikesh, clinicians routinely prescribe aztreonam and piperacillin–tazobactam; however, they also use other antibiotics such as meropenem, cefotaxime, colistin, and levofloxacin in the IPD and EMR department. Future studies can look into the evolution and correlation of antibiotic resistance versus prescription in different departments.

The antibiotic–antibiotic correlation matrix against *P. aeruginosa* indicates an intriguing pattern of interaction among antipseudomonal antibiotics. The colistin–ciprofloxacin combination showed better efficacy (9 out of 17 isolates) than that of colistin–meropenem (4 out of 17 isolates) or colistin–tobramycin (1 out of 17 isolates) in reducing the inhibitory concentration of individual antibiotics below the defined CLSI breakpoint (MIC breakpoint for ciprofloxacin, colistin, meropenem, and tobramycin is ≥4, ≥4, ≥8, and ≥16, respectively) [37]. Furthermore, the ciprofloxacin–meropenem (4 out of 17 isolates) and ciprofloxacin–tobramycin (2 out of 17 isolates) combinations were less effective than the colistin–ciprofloxacin combination. In light of these findings, a close look at pharmacokinetics and pharmacodynamics (PK/PD) of colistin and ciprofloxacin reveals some common characteristics. Both colistin and ciprofloxacin demonstrate concentration-dependent bactericidal activity. Additionally, the therapeutic efficacy of both colistin and ciprofloxacin are dependent on C_max_:MIC ratio [44]. The average plasma concentrations of these two antibiotics were ~5 mg/L within 3 h of administration. Previous reports of antibiotic combinations against *P. aeruginosa* have mixed outcomes. Several studies indicate better efficacy of the colistin–meropenem combination [45,46], while others advocate the colistin–ciprofloxacin combination [47,48]. Our results show that the type of antibiotic interaction is not uniform within the same species (i.e., not all strains of any given species show a specific pattern of antibiotic interaction). Perhaps such a mixed outcome of antibiotic interaction is expected. Antibiotic interaction depends on several factors such as genetic circuits and level of antibiotic resistance, which vary significantly for any given bacterial species [25,49]. However, whether antibiotic interactions show body site-specific or country-specific patterns remains currently unanswered, and it would be interesting to look into it in future studies.

Plasmids play a crucial role in spreading quinolone resistance across clinics and the environment. PMQR offers a selective advantage of hosting cells by providing a basal-level protection against quinolone toxicity and thus enables a favorable evolutionary window for escape. Our data show that plasmids containing quinolone-resistance determinants can be transferred to another bacterial host (here *E. coli* J53) and with a high frequency. Additionally, these plasmids remained stable for an extended period, even without any selection pressure. Indeed, plasmid stability can significantly vary from a few days to several months and even years without any selection pressure [50,51,52]. Plasmid maintenance can be challenging for host cells due to associated fitness costs [53]. However, without a direct selection pressure of antibiotics, plasmid maintenance greatly depends on nutrient availability. Additionally, if the rate of conjugation is sufficient to offset the fitness cost of carriage or loss from segregation, then plasmids may be stable or capable of increasing their frequency in bacterial populations even without any selection pressure [54,55]. Additional molecular mechanisms of such plasmid stability remain to be explored. In this study, we sought to explore the correlation between different types of antibiotic interaction for a given strain. We uncovered two statistically significant correlations; we found a negative correlation between the colistin–ciprofloxacin and the colistin–meropenem combination; similarly, strains showing colistin–meropenem synergy were always positively associated with colistin–tobramycin synergy. Modulation of intracellular concentration of partner antibiotics may partially explain this phenomenon, as it has been observed for many membrane-active compounds [56,57]. However, sometimes a membrane-active compound can also interfere with antibiotic import and thus decrease the intracellular concentration of the partner antibiotic; this has been seen with benzalkonium chloride (a membrane acting agent) interfering with the import of ciprofloxacin and gentamicin in *E. coli* [25].

The intrinsic resistance of *P. aeruginosa* against multiple biocides is largely contributed by efflux pumps [58]. Efflux pumps do not only play a pivotal role in providing the MDR phenotype but are also required for transporting virulence factors and signaling molecules [39]. Corroborating previous studies, our results show the basal level difference in activity of efflux pumps between resistant and susceptible isolates (Figure 5) [40,59]. Notably, due to heavy dependence on efflux pumps, targeting resistant isolates with membrane-active agents like colistin may show a proportionally higher effect than susceptible isolates.

Our study provided some new insights into synergistic interactions among antipseudomonal antibiotics. However, the current study has some limitations. First, the clinical isolates used in the study only represents a specific geographical location, and future studies may include isolates from different locations around the world. Second, we did not compare long-term evolutionary aspects of the combinations with respect to the generation of resistant mutants, and this would be interesting to look into it. Despite their growing biomedical significance, central questions about drug interactions remain unanswered; specifically, little is known about the underlying mechanisms of most drug interactions [60]. Overall, our data suggest that antibiotic interactions show a complex pattern. Several factors such as type of antibiotic resistance, presence, and level of expression of efflux pumps, plasmids, and nutritional status might contribute to it. Systemic identification of antibiotic interaction could provide us valuable insights and guide us in selecting appropriate antibiotic regimens for the emerging threat of MDR and XDR *P. aeruginosa* infections.

## 4. Materials and Methods

### 4.1. Chemicals and Biological Materials

All the bacteriological media were purchased from HIMEDIA, India. All antibiotics powder used in this study was obtained from Sigma Aldrich, St. Louis, MO, USA, while antibiotic discs were obtained from HIMEDIA, India. All clinical strains of *P. aeruginosa* were isolated from any of the following sources from patients with bacterial infections who were admitted to All India Institute of Medical Sciences Rishikesh, India between April 2018 to March 2019 (Supplementary Table S1): blood, urine, pus, pleural fluids, sputum, endotracheal aspirate, bronchoalveolar lavage, or infected tissue. Bacterial species other than *P. aeruginosa* were also isolated from infected samples; however, they are not part of this study. Clinical strains were routinely cultured on 5% sheep blood agar. Species identification of all causative microorganisms was performed using either Bruker’s MALDI Biotyper^®^ Microbial Identification system (Bruker, Billerica, MA, USA) or MicroScan WalkAway 96 *Plus* ID/AST System (Bechman Coulter, Brea, CA, USA) as per manufacturer’s recommendation. *E. coli* J53 was obtained from the Department of Bacteriology, Postgraduate Institute of Medical Education and Research, Chandigarh.

### 4.2. Antibiotics Susceptibility Assay

Initial antibiotic susceptibility assay of all clinical strains was performed using an automated antibiotic susceptibility testing system (MicroScan WalkAway 96 *Plus* ID/AST System, Bechman Coulter, USA). Additionally, the susceptibility of 243 identified *P. aeruginosa* strains against 15 antipseudomonal antibiotics was also determined using the disk diffusion method according to CLSI recommendation [37]. For 17 extensively drug-resistance *P. aeruginosa* strains (Supplementary Table S1), minimum inhibitory concentration (MIC) values of the studied antimicrobials were determined using 2-fold broth microdilution method in a 96-well polystyrene plate with an initial inoculum of 10^6^ CFU/mL in Cation adjusted Mueller Hinton broth (CAMHB). Antibiotic susceptibility results were interpreted according to the guidelines recommended by the Clinical and Laboratory Standards Institute (CLSI), USA [37]. Seventeen *P. aeruginosa* strains were selected based on the presence of PMQR (please refer to Section 4.4) and XDR phenotype.

### 4.3. Screening of Antibiotic-Antibiotic Combinations

In order to determine the best suitable antibiotic pairs for the treatment of MDR *P. aeruginosa* strains, four major antipseudomonal antibiotics, i.e., polymyxin B, tobramycin, meropenem, and ciprofloxacin (representing different antibiotic classes—polymyxins, aminoglycosides, carbapenems, and fluoroquinolones) were probed to each other using a two-dimensional checkerboard assay. Two-dimensional checkerboard assay was performed according to the previously described method [49]. The fractional inhibitory concentration index (FICI) of antibiotic pairs was determined to evaluate the type of drug interaction. Synergy was defined if the drug pair had an FICI value of ≤0.5, and additivity was defined as an FICI value of ≥0.5 to <4, whereas antagonism was defined as an FICI value of ≥4 [61].

### 4.4. Identification of Quinolone Resistance Determinants

Chromosomal and plasmid-mediated resistance determinants have been reported for quinolone resistance in Gram-negative bacteria. Hence, presence of chromosomal and plasmid-mediated quinolones resistance determinants (*qnrA*, *qnrB*, *qnrD*, *qnrS*, *qepA*, *aqxA*, *aqxB*, *aac(6′)-Ib-cr*, *gyrA*, *gyrB*, *parC*, *parE*) were checked using PCR. PCR conditions included an initial denaturation at 95 °C for 1 min followed by 30 cycles of 95 °C for 30 s and annealing for 30 s at 54 °C for 12 genes with an extension at 72 °C for 30 s. Cycling was followed by a final extension at 72 °C for 5 min. A complete list of primers used for PCR amplification along with the product sizes is provided in Table 1.

### 4.5. Bacterial Conjugation

Conjugation experiments were performed with six MDR *P. aeruginosa* strains as donors and *E. coli* J53 (azide-resistant) as the recipient in LB broth [69]. Briefly, donor and recipient cells were grown in LB broth to O.D._600nm_~0.5. Conjugation was performed by mixing donor and recipient cells in a 1:1 ratio in LB broth followed by incubation at 37 °C for 12 h without shaking. Transconjugants were selected on LB agar plates co-supplemented with sodium azide (100 mg/L; Sigma-Aldrich, USA) and ciprofloxacin (4 mg/L) for counterselection of plasmid-encoded quinolone-resistant determinants. Transconjugation frequencies were calculated by dividing the number of transconjugants by the number of donor cells. The conjugation experiments were performed with three biological replicates.

### 4.6. Measurement of Plasmid Stability and Bacterial Fitness

To check the stability of inter-species plasmid transfer, transconjugants (*E. coli* J53 having plasmids) were serially passaged daily on antibiotic-free LB agar plates for ten days. For positive control, transconjugants were serially passaged on LB agar supplemented with ciprofloxacin. The presence of respective plasmid-mediated resistance determinants was assessed using PCR on the 5th and 10th days. For analyzing bacterial fitness, the growth of transconjugants and *E. coli* J53 (wild type) in antibiotic-free medium (LB medium) was compared by measuring optical density at 600 nm. Briefly, transconjugant and *E. coli* J53 cells grown overnight were incubated in fresh LB medium each at a starting O.D._600nm_~0.05. Growth was measured using a spectrophotometer at an interval of 30 min for 16 h.

### 4.7. Ethidium Bromide Accumulation Assay

Ethidium bromide is a heterocyclic compound and a common substrate for major efflux pumps in Gram-negative bacteria. Ethidium bromide accumulation assay was performed as previously described [70,71]. *P. aeruginosa* cells were grown to an O.D._600nm_~0.6, followed by washing with PBS, and finally resuspended in PBS to an O.D._600nm_~0.2. Cells were loaded with 20 mg/L of ethidium bromide and 0.4% glucose (wt./vol) and were immediately aliquoted into a black 96-well plate (Fluorescence BRANDplates^®^, Essex, CT, USA). Fluorescence was measured using a Tecan Infinite^®^ 200 PRO fluorescence spectrophotometer (Tecan, Männedorf, Switzerland) at an excitation wavelength of 520 nm and an emission wavelength of 590 nm for 60 min every 4 min.

## Figures and Tables

**Table 1:** Table describing target genes and primers used for detecting PMQR (plasmid mediated quinolone resistance genes) and QRDR (chromosomal quinolone resistance determinants regions).

Target Gene	Nucleotide Sequence (5’-3’)	Product Size	References
*qnrA*	F-5′-AGAGGATTTCTCACGCCAGG-3’	580	[62]
	R-5′-TGCCAGGCACAGATCTTGAC-3’		
*qnrB*	F-5′-GGCATTGAAATTCGCCACTG-3’	263	[62]
	R-5′-TTTGCTGCTCGCCAGTCGAA-3’		
*qnrD*	F-5′-CGAGATCAATTTACGGGGAATA-3’	533	[63]
	R-5′-AACAAGCTGAAGCGCCTG-3’		
*qnrS*	F-5′-GCAAGTTCATTGAACAGGGT-3’	428	[62]
	R-5′-TCTAAACCGTCGAGTTCGGCG-3’		
*qepA*	F-5′-AACTGCTTGAGCCCGTAGAT-3’	596	[64]
	R-5′-GTCTACGCCATGGACCTCAC-3’		
*oqxA*	F-5′-CTCGGCGCGATGATGCT-3′	392	[65]
	R-5′-CCACTCTTCACGGGAGACGA-3′		
*oqxB*	F-5′-TTCTCCCCCGGCGGGAAGTAC-3′	512	[65]
	R-5′-CTCGGCCATTTTGGCGCGTA-3′		
*acc(6’)-Ib-cr*	F-5′-TTGCGATGCTCTATGAGTGGCTA-3’	482	[66]
	R-5′-GTCTACGCCATGACCTCAC-3’		
*gyrA*	F-5’-GTGTGCTTTATGCCATGAG-3’	287	[67]
	R-5’-GGTTTCCTTTTCCAGGTC-3’		
*gyrB*	F-5’-GCGGTGGAACAGGAGATGGGCAAGTAC-3’	510	[68]
	R-5’-CTGGCGGAAGAAGAAGGTCAACA-3’		
*parC*	F-5’-CGAGCAGGCCTATCTGAACTAT-3’	357	[68]
	R-5’-AGCAGCACCTCGGAATAG-3’		
*parE*	F-5’-CTGGCGGAAGAAGAAGGTCAACA-3’	592	[68]
	R-5’-TCGAGGGCGTAGTAGATGTCCTTGCCG-3’		

## Data Availability

Data is contained within the article and supplementary material.

## Supplementary Materials

The following supporting information can be downloaded at: https://www.mdpi.com/article/10.3390/ph15020243/s1, Figure S1: Heatmap showing QRDR & PMQR; Table S1: Strains used in the study.
