# Supplementary material for: Potential Synergistic Antibiotic Combinations against Fluoroquinolone-Resistant Pseudomonas aeruginosa
Source: Pharmaceuticals (Basel). 2022 Feb 18;15(2):243. doi: 10.3390/ph15020243 (PMC8880063; doi:10.3390/ph15020243)
Supplement: Supplementary file 1 [file pharmaceuticals-15-00243-s001.zip › pharmaceuticals-1585108-supplementary.pdf]

Supplementary Material

Potential Synergistic Antibiotic Combinations against Fluoroquinolone-Resistant *Pseudomonas aeruginosa*

Ashish Kothari [1], Neeraj Jain [2,3], Shyam Kishor Kumar [4], Ankur Kumar [1], Karanvir Kaushal [5], Satinder Kaur [6], Atul Pandey [7,8,*], Amit Gaurav [9,*] and Balram Ji Omar [1,*]

[1] Department of Microbiology, All India Institute of Medical Sciences Rishikesh, Rishikesh 249203, India; ash-ish.sci@aiimsrishikesh.edu.in (Ashish Kothari); ankurk591@gmail.com (Ankur Kumar)
[2] Department of Medical Oncology, All India Institute of Medical Sciences Rishikesh, Rishikesh 249203, India; neeraj.monc@aiimsrishikesh.edu.in
[3] Division of Cancer Biology, Central Drug Research Institute, Lucknow 226031, India
[4] Department of Microbiology, All India Institute of Medical Sciences Deoghar, Deoghar 814152, India; dr.shyamkishor84@gmail.com
[5] Department of Biochemistry, All India Institute of Medical Sciences Rishikesh, Rishikesh 249203, India; karanvirkaushal@gmail.com
[6] Department of Microbiology, Postgraduate Institute of Medical Education and Research, Chandigarh 160012, India; satinder.silky20@gmail.com
[7] Department of Ecology, Evolution and Behavior, The Hebrew University of Jerusalem, Jerusalem 9190401, Israel
[8] Department of Ecology and Evolutionary Biology, University of Michigan, Ann Arbor, MI 48109, USA
[9] Satbiome, Dehradun 248007, India
* Correspondence: atulkumarpandey@gmail.com (A.P.); amitgaurav@satbiome.com (A.G.); balram.micro@aiimsrishikesh.edu.in (B.J.O.); Tel.: +91-9415026640 (B.J.O.)

Supplementary Table S1. Table showing strain ID, source of isolation and antibiotic resistance pattern against 15 antipseudomonal antibiotics. PTZ – Piperacillin/tazobactam, CAZ – Ceftazidime, CPM – Cefepime, AT – Aztreonam, IMP – Imipenem, MRP – Meropenem, DOR – Doripenem, AK – Amikacin, GEN – Gentamicin, TOB – Tobramycin, CIP – Ciprofloxacin, LE – Levofloxacin, OF – Ofloxacin, CL – Colistin, COT – Co-trimoxazole. Strain ID with * highlight strains for which minimum inhibitory concentrations were also measured using 2-fold broth microdilution method according to CLSI method [37].

S. No.	Strain ID	Source	PTZ	CAZ	CPM	AT	IMP	MRP	DOR	AK	GEN	TOB	CIP	LE	OF	CL	COT
**1**	1916	Blood	R	R	R	R	R	R	R	R	R	R	R	R	R	S	R
**2**	1566	Blood	S	S	S	S	S	S	S	S	S	S	S	S	S	S	S
**3**	1826	Blood	S	R	R	R	S	S	S	R	R	R	R	R	S	S	S
**4**	1827	Blood	S	R	R	R	S	S	S	R	R	R	R	R	I	S	R
**5**	1944	Blood	S	S	S	S	S	S	S	S	S	S	S	S	S	S	S
**6**	4324	Blood	S	S	S	S	S	S	S	R	S	S	S	S	S	S	S
**7**	4426	Blood	R	R	R	R	S	R	S	R	S	R	S	S	S	S	R
**8**	4595	Blood	S	S	S	S	S	S	S	R	R	S	S	S	S	S	S
**9**	4663*	Blood	R	R	R	R	R	R	R	R	R	R	R	R	R	R	R
**10**	10525	Urine	S	R	S	S	R	S	R	R	R	S	R	R	S	S	S
**11**	11965	Urine	S	R	R	S	S	S	R	R	S	R	R	R	R	S	R
**12**	10608	Urine	R	R	S	R	R	R	R	R	R	R	R	R	R	S	R
**13**	11016	Urine	R	R	R	R	R	R	R	R	R	R	R	R	I	S	S
**14**	11334	Urine	R	R	R	R	R	R	R	R	R	R	R	S	S	S	R
**15**	11521	Urine	S	S	S	S	R	S	R	S	S	S	R	R	R	S	S
**16**	11553	Urine	S	R	S	R	R	R	R	R	S	S	R	I	S	S	S

**17**	11729	Urine	S	R	S	S	S	S	S	S	R	R	S	S	S	S	S
**18**	5679	Urine	R	R	R	S	R	R	R	R	R	R	R	R	R	S	R
**19**	5749	Urine	S	S	R	S	R	S	R	R	R	R	R	R	R	S	S
**20**	5830	Urine	S	S	S	S	R	S	R	S	R	R	R	R	R	S	S
**21**	5921	Urine	S	S	S	S	S	S	S	S	S	S	S	S	S	S	S
**22**	6433	Urine	S	S	R	S	R	S	R	R	R	R	R	R	R	S	S
**23**	6817	Urine	R	R	R	R	R	R	R	R	R	R	R	S	S	S	R
**24**	6964	Urine	S	R	R	S	R	S	R	R	R	R	R	R	R	S	S
**25**	7286	Urine	S	R	R	S	R	S	R	R	R	R	R	S	S	S	S
**26**	7360	Urine	S	S	R	S	R	S	R	R	R	R	R	R	S	S	S
**27**	8447	Urine	S	R	R	R	R	S	R	R	R	R	R	R	R	S	S
**28**	8969	Urine	S	S	S	S	S	S	S	S	S	S	S	S	S	S	S
**29**	9430	Urine	R	R	R	R	R	R	R	R	R	R	R	R	R	S	R
**30**	9975	Urine	S	R	S	R	R	S	R	R	R	R	R	R	R	S	R
**31**	8525	Pus	S	R	S	S	R	S	R	S	R	S	R	R	S	S	S
**32**	10537*	Urine	R	R	R	R	R	R	R	S	R	R	R	R	R	S	S
**33**	10185	Pleural Fluid	R	R	S	R	R	R	R	S	R	S	R	R	R	S	R
**34**	8617	Pus	S	R	R	R	R	R	R	S	R	S	R	S	S	S	S
**35**	9864	Pus	R	R	R	R	R	R	R	S	R	R	R	R	R	S	R
**36**	10199	Sputum	S	S	S	S	R	S	R	S	S	R	R	R	R	S	S
**37**	10252	Percutaneous Nephrostomy	S	R	R	R	R	R	R	R	S	S	R	R	S	S	I

**38**	11729(D)	Urine	S	R	S	S	S	S	S	S	R	S	S	S	S	S	S
**39**	4609	Urine	S	S	S	S	R	S	R	S	R	R	R	R	S	S	I
**40**	4898	Urine	R	R	R	S	R	R	R	R	R	R	R	R	R	S	R
**41**	4902	Urine	I	R	R	S	R	S	R	R	R	R	R	S	S	S	S
**42**	4985	Urine	S	S	S	S	S	S	S	S	S	S	S	S	S	S	S
**43**	5284	Urine	S	S	S	S	R	S	R	S	R	S	R	R	R	S	R
**44**	5353	Urine	S	R	R	S	R	R	R	R	R	R	R	R	R	S	R
**45**	2189	Pus	S	S	S	S	S	S	S	S	S	S	S	S	S	S	S
**46**	2191	Pus	R	R	R	S	R	R	R	R	R	R	R	R	R	S	R
**47**	2277(D)	Pus	S	S	S	S	S	S	S	S	S	S	S	S	S	S	S
**48**	2284(D)*	Pus	R	R	R	R	R	R	R	R	R	R	R	R	R	R	R
**49**	2302(D)	Endotracheal aspirates	R	R	R	R	R	R	R	R	R	R	R	R	R	S	R
**50**	2407(D)	Endotracheal aspirates	R	R	R	R	R	R	R	R	R	R	R	R	R	S	R
**51**	2487*	Pus	R	R	R	R	R	R	R	R	R	R	R	R	R	R	R
**52**	2566	Pus	S	S	S	S	R	S	R	S	R	R	R	R	R	S	R
**53**	2606	Sputum	S	R	R	S	R	R	R	R	R	R	R	R	R	S	R
**54**	2640	Pus	S	S	R	S	R	S	R	S	R	R	R	R	S	S	S
**55**	2679	Sputum	S	R	S	S	R	S	R	S	R	R	R	S	S	S	S
**56**	2742	Sputum	S	S	S	S	S	S	S	S	S	S	S	S	S	S	S
**57**	2899	Pus	R	R	R	R	R	R	R	R	R	R	R	R	S	S	R
**58**	2931	Pus	S	S	S	S	S	S	S	S	S	S	S	S	S	S	S

**59**	3015	Endotracheal aspirates	R	R	R	R	R	R	R	R	R	R	R	R	R	S	R
**60**	3067	Pus	S	S	S	S	S	S	S	S	S	S	S	S	S	S	S
**61**	3142	Pus	S	R	R	R	R	R	R	S	R	R	R	R	S	S	R
**62**	3149	Pus	S	S	S	S	R	S	R	S	S	S	R	R	R	S	R
**63**	3173*	Endotracheal aspirates	R	R	R	R	R	R	R	R	R	R	R	R	R	R	R
**64**	3209	Pus	S	S	S	S	S	S	S	S	S	S	S	S	R	S	R
**65**	3260	Pus	S	S	S	S	S	S	S	S	S	S	S	S	S	S	S
**66**	3294	Endotracheal aspirates	S	S	S	S	R	S	R	S	S	S	S	S	S	S	S
**67**	3300	Endotracheal aspirates	S	S	S	S	S	S	S	S	S	S	S	S	S	S	S
**68**	3621	Pus	S	S	R	S	R	S	R	R	R	R	R	R	S	S	S
**69**	3725	Pus	S	S	S	S	R	S	R	R	R	R	R	S	S	S	S
**70**	3736	Sputum	S	S	S	S	S	S	S	S	S	S	S	S	S	S	S
**71**	3738	Bronchoalveolar Lavage	S	R	S	R	R	S	R	R	S	S	R	S	S	S	S
**72**	3751*	NA	R	R	R	R	R	R	R	R	R	R	R	R	R	R	R
**73**	3796	Bronchoalveolar Lavage	S	R	S	S	R	S	R	S	R	S	R	S	S	S	S
**74**	3852	Pus	S	S	S	S	S	S	S	R	R	R	R	S	S	S	S
**75**	3881	Pus	S	S	S	S	S	S	S	S	S	S	S	S	S	S	S
**76**	4039	Pus	S	S	S	S	S	S	S	S	S	S	S	S	S	S	S
**77**	4097	Sputum	S	R	R	R	R	S	R	R	R	R	R	R	S	S	S
**78**	4163	Sputum	S	S	S	S	S	S	S	S	S	S	S	S	S	S	S
**79**	4220	Sputum	S	R	R	R	R	R	R	R	R	R	R	R	S	S	S

**80**	4238	Pus	S	R	S	S	S	S	S	S	S	S	R	S	S	S	S
**81**	4293	Pus	S	R	R	R	R	R	R	R	R	R	R	R	S	S	S
**82**	4363	Pus	S	R	R	S	R	S	R	R	R	R	R	R	R	S	R
**83**	4415	Sputum	S	R	R	R	R	S	R	S	S	R	R	R	S	S	S
**84**	4563	Bronchoalveolar Lavage	S	S	S	S	S	S	S	S	S	S	S	S	S	S	S
**85**	4621	Bronchoalveolar Lavage	S	S	S	S	S	S	S	S	R	R	R	S	S	S	S
**86**	4634	Pus	S	S	R	R	R	S	R	R	R	S	R	R	S	S	S
**87**	4645	Sputum	S	S	S	S	S	S	S	S	S	S	S	S	S	S	S
**88**	4703	Pus	S	R	R	R	R	S	R	R	R	R	R	R	R	S	R
**89**	4712	Pus	S	S	S	S	S	S	S	S	S	S	S	S	S	S	S
**90**	4780	NA	S	S	S	S	S	S	S	S	S	S	S	S	S	S	S
**91**	4786*	Pus	R	R	R	R	R	R	R	R	R	R	R	R	R	R	R
**92**	4878	Pus	S	S	S	S	S	S	S	R	R	R	S	I	S	S	S
**93**	4882	Bronchoalveolar Lavage	S	S	S	S	S	S	S	S	S	S	S	S	S	S	S
**94**	4931	Sputum	S	S	S	S	S	S	S	S	S	S	S	S	S	S	S
**95**	4963	Pus	S	R	R	R	R	S	R	R	R	R	R	S	S	S	S
**96**	4979	Pus	S	S	S	S	S	S	S	S	S	S	S	S	S	S	S
**97**	5036	Pus	S	S	S	S	S	S	S	S	S	S	S	S	S	S	S
**98**	5073	Sputum	S	R	R	R	R	I	R	R	R	R	R	R	S	S	S
**99**	5233	Sputum	S	S	S	S	S	S	S	S	S	S	S	S	S	S	S
**100**	5268	Endotracheal aspirates	S	S	S	S	S	S	S	S	S	S	S	S	S	S	S

**101**	5301	Sputum	S	S	I	I	I	S	I	S	S	S	S	S	S	S	S
**102**	5319	Pus	S	S	I	S	S	S	S	S	S	S	S	S	S	S	S
**103**	5396	Pus	S	R	I	S	R	S	R	R	R	R	R	S	S	S	S
**104**	5416	Sputum	S	R	S	S	R	R	R	R	R	R	R	R	S	S	S
**105**	5532	Sputum	S	R	S	R	R	S	R	R	R	R	R	R	R	S	S
**106**	5543	Sputum	S	S	S	S	S	S	S	S	S	S	S	S	S	S	S
**107**	5569	Pus	S	S	S	S	S	S	S	S	S	S	S	S	S	S	S
**108**	5570	Pus	S	S	S	S	S	S	S	S	S	S	S	S	S	S	S
**109**	5574	Endotracheal aspirates	R	R	S	S	S	R	S	S	S	S	S	S	S	S	S
**110**	5608	Sputum	R	R	S	R	R	R	R	R	R	R	R	R	R	S	S
**111**	5623*	Pus	R	R	R	R	R	R	R	R	R	R	R	R	R	R	R
**112**	5634	Tracheostomy tube secretion	R	R	R	R	R	R	R	R	R	R	R	R	R	R	R
**113**	5647	Sputum	S	S	R	S	S	S	S	R	R	S	S	S	S	S	S
**114**	5665	Pus	S	R	R	R	S	S	S	R	R	R	R	R	R	S	R
**115**	5674*	Sputum	R	R	R	R	R	R	R	R	R	R	R	R	R	R	R
**116**	5685	Sputum	S	R	S	S	S	S	S	S	S	S	S	S	S	S	S
**117**	5741	Sputum	S	S	S	S	S	S	S	S	S	S	S	S	S	S	S
**118**	5831	Sputum	S	S	S	S	S	S	S	S	S	S	S	S	S	S	S
**119**	5863	Pus	S	S	S	S	S	S	S	S	S	S	S	S	S	S	S
**120**	5918	Sputum	S	S	S	R	R	S	R	S	R	R	R	R	R	S	S
**121**	5978*	Sputum	R	R	R	R	R	R	R	R	R	R	R	R	R	R	R

**122**	5994	Endotracheal aspirates	S	S	S	S	S	S	S	S	S	S	S	S	S	S	S
**123**	6003	Tracheostomy tube secretion	S	R	R	R	R	S	R	R	R	R	S	S	R	S	S
**124**	6019	Endotracheal aspirates	S	S	S	S	S	S	S	S	S	S	S	S	S	S	S
**125**	6022	Sputum	S	S	S	S	S	S	S	S	S	S	S	S	S	S	S
**126**	6097	Pigtail Fluid	S	R	R	S	R	R	R	R	S	S	S	S	S	S	S
**127**	6373	Tracheostomy tube secretion	S	S	S	S	S	S	S	S	S	S	S	S	S	S	S
**128**	6467	Sputum	S	S	S	S	S	S	S	S	S	S	S	S	S	S	S
**129**	6469	Tracheostomy tube secretion	S	S	S	S	S	S	S	S	S	S	S	S	S	S	S
**130**	6514	Pleural Fluid	S	R	R	R	S	R	S	R	S	R	S	R	R	S	R
**131**	6518	Pus	S	S	S	S	S	S	S	S	S	S	S	S	S	S	S
**132**	6535	Tissue	S	S	S	S	S	S	S	S	S	S	S	S	S	S	S
**133**	6546	Pus	S	S	S	S	S	S	S	S	S	S	S	S	S	S	S
**134**	6551	Pus	R	R	S	R	S	S	S	S	S	S	S	S	S	S	S
**135**	6553	Pus	S	R	S	R	S	S	S	S	S	S	S	S	S	S	S
**136**	6555	Pleural Fluid	S	R	S	R	S	S	S	S	S	S	S	S	S	S	S
**137**	6567	Tracheostomy tube secretion	S	R	R	S	R	S	R	S	R	R	R	R	S	S	S
**138**	6634	Pus	S	R	R	S	R	S	R	S	S	S	R	R	S	S	S
**139**	6668	Endotracheal aspirates	S	S	S	S	S	S	S	S	S	S	S	S	S	S	S
**140**	6711	Pus	S	S	S	S	R	S	R	S	R	R	R	S	S	S	S
**141**	6716	Sputum	R	R	R	R	R	R	R	R	R	R	R	R	R	S	R
**142**	6728	Pus	S	R	S	S	R	S	S	S	R	R	R	R	S	S	S

**143**	6769	Tracheostomy tube secretion	S	R	R	R	R	S	S	R	R	R	R	R	S	S	S
**144**	6825	Endotracheal aspirates	S	R	R	S	R	R	R	R	R	R	R	R	S	S	S
**145**	6837	Pus	S	S	S	S	S	S	S	S	S	S	R	S	S	S	S
**146**	6895	Pleural Fluid	S	R	R	R	S	S	S	S	R	S	S	S	R	S	R
**147**	6923	Pigtail Fluid	S	I	S	S	S	R	R	R	S	R	S	R	S	S	S
**148**	6926	Endotracheal aspirates	S	S	S	S	S	S	R	S	S	S	S	S	S	S	S
**149**	6950	Pleural Fluid	S	R	S	R	R	S	R	S	R	R	R	R	S	S	S
**150**	6954*	Endotracheal aspirates	R	R	R	R	R	R	R	R	R	R	R	R	R	R	R
**151**	6982	Endotracheal aspirates	S	R	S	S	R	R	R	R	S	S	R	S	R	S	R
**152**	7001*	Pus	R	R	R	R	R	R	R	R	R	R	R	R	R	R	R
**153**	7093	Bronchoalveolar Lavage	S	S	S	S	S	S	S	S	S	S	S	S	S	S	S
**154**	7204	Tracheostomy tube secretion	S	S	S	S	S	S	S	S	S	S	S	S	S	S	S
**155**	7236	Pus	S	S	S	S	S	S	R	S	R	S	S	S	S	S	S
**156**	16078	Blood	R	R	R	R	R	R	R	R	R	R	I	R	R	I	R
**157**	16922	Urine	S	R	R	S	R	R	R	R	R	R	R	R	R	S	R
**158**	10723	Urine	S	S	S	S	S	S	S	S	S	S	S	S	S	S	S
**159**	11085	Urine	S	S	S	S	R	S	R	S	S	S	R	R	R	S	S
**160**	11141	Urine	S	R	S	S	R	S	R	S	R	R	R	S	S	S	S
**161**	11292	Urine	S	S	S	R	R	S	R	S	R	S	R	R	R	S	S
**162**	11747	Urine	S	S	S	S	R	S	R	S	R	S	R	R	R	S	S
**163**	5715*	Urine	R	R	R	R	R	R	R	R	R	R	R	R	R	R	R

**164**	5721	Urine	S	S	S	S	S	S	S	S	S	S	S	S	S	S	S
**165**	6426	Urine	S	S	S	S	S	S	S	S	S	S	S	S	S	S	S
**166**	6987	Urine	S	S	R	S	R	S	R	R	S	R	R	R	R	S	S
**167**	7295	Urine	R	R	R	R	R	R	R	R	R	R	R	R	S	S	S
**168**	7493	Urine	S	R	R	S	R	S	R	R	R	R	R	R	R	S	S
**169**	7915	Urine	S	S	S	S	R	S	R	S	S	S	R	S	S	S	S
**170**	8541	Urine	S	R	R	R	R	S	R	R	R	R	R	S	S	S	S
**171**	8679	Urine	S	S	R	S	R	S	R	R	R	R	R	R	R	S	S
**172**	8900	Urine	S	S	R	S	R	S	R	R	R	R	R	R	R	S	S
**173**	8927*	Urine	R	R	R	R	R	R	R	R	R	R	R	R	R	R	R
**174**	9961	Urine	S	R	S	S	R	S	R	R	R	R	R	R	R	S	R
**175**	10302	Urine	R	R	R	R	R	R	R	R	R	S	R	R	R	S	R
**176**	11723	Urine	S	S	S	S	S	S	S	R	S	R	S	S	S	S	S
**177**	18363	Percutaneous Nephrostomy	S	S	S	S	R	S	R	R	S	R	R	R	R	S	S
**178**	17809	Urine	S	R	S	S	R	S	R	S	R	S	R	R	S	S	S
**179**	17803	Urine	S	S	S	R	R	S	R	S	R	S	R	R	R	S	S
**180**	17800	Urine	S	S	S	S	R	S	R	R	R	S	R	R	R	S	S
**181**	3848	Urine	S	S	R	S	R	S	R	S	R	R	R	R	R	S	I
**182**	3867	Urine	S	S	R	S	R	S	R	S	R	R	R	S	S	S	S
**183**	4036	Urine	S	S	S	S	S	S	S	S	S	S	S	S	S	S	S
**184**	4185	Urine	S	S	S	S	S	S	S	S	S	S	S	S	S	S	S

**185**	4200	Urine	S	R	R	S	R	S	R	S	R	R	R	R	S	S	I
**186**	4757*	Urine	R	R	R	R	R	R	R	R	R	R	R	R	R	R	R
**187**	4834	Urine	S	R	R	S	R	S	R	S	R	R	R	R	R	S	S
**188**	2211	Sputum	S	S	S	S	S	S	S	S	R	S	S	S	S	S	S
**189**	2412	Pus	S	S	S	S	S	S	S	S	S	S	S	S	S	S	S
**190**	2428	Pus	S	S	S	S	S	S	S	S	S	S	S	S	S	S	S
**191**	2458	Sputum	S	S	S	S	S	S	S	S	S	S	S	S	S	S	S
**192**	2460	Sputum	S	S	S	S	S	S	S	S	S	S	S	S	S	S	S
**193**	2530	Knee aspirate	S	R	R	S	R	S	R	S	R	R	R	R	S	S	S
**194**	2538	Pus swab	S	S	S	S	R	S	R	S	S	S	S	S	S	S	S
**195**	2725	Pus	S	S	S	S	S	S	S	S	S	S	S	S	S	S	S
**196**	2728	Pus	S	S	S	S	S	S	S	S	S	S	S	S	S	S	S
**197**	2751	Ear swab	S	S	S	S	R	S	R	S	R	R	S	S	S	S	S
**198**	2839	Sputum	S	S	S	S	S	S	S	S	S	S	S	S	S	S	S
**199**	3136	Pus	S	S	S	S	S	S	S	S	S	S	S	S	S	S	S
**200**	3314	Pus	S	S	S	S	R	S	R	S	R	R	R	R	R	S	R
**201**	3780	Pus	S	S	R	S	S	S	S	S	S	S	R	S	S	S	S
**202**	3782	Pus	S	S	R	S	R	S	R	R	R	R	R	R	S	S	S
**203**	3845	Pus	R	R	R	S	S	S	S	R	R	R	R	R	S	S	S
**204**	4000	Bronchoalveolar Lavage	S	S	S	S	R	S	R	S	S	S	S	S	S	S	S
**205**	4187	Bronchoalveolar Lavage	S	S	S	S	S	S	S	S	S	S	S	S	S	S	S

**206**	4252	Sputum	S	S	S	S	S	S	S	S	S	S	S	S	S	S	S
**207**	4304	Expressed prostatic secretions	S	S	S	S	S	S	S	S	S	S	S	S	S	S	S
**208**	4333	Pus	S	S	S	S	R	S	R	S	S	S	R	R	R	S	R
**209**	4380	Pus	S	S	S	S	S	S	S	S	S	S	S	S	S	S	S
**210**	4610	Tissue	S	R	R	S	S	S	S	R	R	R	S	S	S	S	S
**211**	4658	Pus	S	S	R	S	R	S	R	R	R	R	R	R	S	S	S
**212**	5159	Tissue	S	R	S	R	R	R	R	R	R	R	R	R	R	S	S
**213**	5207	Knee aspirate	S	S	S	S	S	S	S	S	S	S	S	S	S	S	S
**214**	5399	Tissue	S	S	S	S	S	S	S	S	S	S	S	S	S	S	S
**215**	5447	Tissue	S	R	S	R	R	S	R	R	R	R	R	R	S	S	S
**216**	5719	Pus	S	R	S	S	R	S	R	R	R	R	R	R	R	S	R
**217**	5753	Sputum	S	S	S	S	S	S	S	S	S	S	S	S	S	S	S
**218**	5769	Pus	S	S	S	S	S	S	S	S	S	S	S	S	S	S	S
**219**	5770	Pus	S	S	S	S	R	S	R	R	R	R	R	R	R	S	S
**220**	6374	Pus	S	S	S	S	S	S	S	S	S	S	S	S	S	S	S
**221**	6552	Pus	S	S	S	S	S	S	S	S	S	S	S	S	S	S	S
**222**	6888	Pus	S	R	S	R	S	S	S	S	R	R	R	R	S	S	S
**223**	6965	Sputum	S	S	S	S	S	S	R	S	S	S	S	S	S	S	S
**224**	AIIMS-224	Pus	R	S	R	R	R	R	I	R	R	R	R	R	R	S	R
**225**	5824	Urine	S	S	R	S	R	S	R	R	R	R	R	S	S	S	S
**226**	6447	Urine	S	S	R	S	R	S	R	R	R	R	R	R	R	S	S

**227**	7133	Urine	S	R	R	S	R	S	R	R	R	R	R	R	R	S	S
**228**	9015	Urine	S	R	R	S	R	S	R	R	S	R	R	R	R	S	S
**229**	3678*	Pus	R	R	R	S	R	R	R	R	R	R	R	R	R	R	R
**230**	3769	Bronchoalveolar Lavage	S	R	R	R	R	R	R	R	R	R	R	R	S	S	S
**231**	3851	Sputum	S	R	S	S	R	S	R	S	S	S	R	S	S	S	S
**232**	3887	Endotracheal aspirates	S	R	R	R	R	R	R	S	R	R	R	R	S	S	S
**233**	3956*	Pus	R	R	R	R	R	R	R	R	R	R	R	R	R	R	R
**234**	4082	Endotracheal aspirates	R	S	R	S	S	R	S	R	S	R	R	R	S	S	S
**235**	4103	Pus	I	R	R	S	R	R	R	R	R	R	R	R	R	S	R
**236**	4112	Ascitic Fluid	S	S	R	S	R	S	R	R	R	R	R	R	S	S	S
**237**	4263	Pus	S	S	R	S	R	R	R	S	R	R	R	R	S	S	S
**238**	4286	Tracheostomy tube secretion	S	R	R	R	R	R	R	R	R	R	R	R	S	S	S
**239**	4769	Endotracheal aspirates	S	R	R	R	R	R	R	R	R	R	R	R	R	S	R
**240**	5821	Endotracheal aspirates	S	R	S	S	R	R	R	R	R	R	R	R	R	S	R
**241**	6401	Pus	S	S	S	S	R	S	R	S	S	S	R	S	S	S	S
**242**	6440	Pus	S	R	R	S	R	S	R	R	R	R	R	R	R	S	R
**243**	6524	Pus	R	R	R	R	R	R	R	R	R	R	R	R	R	R	R

Supplementary Figure S1

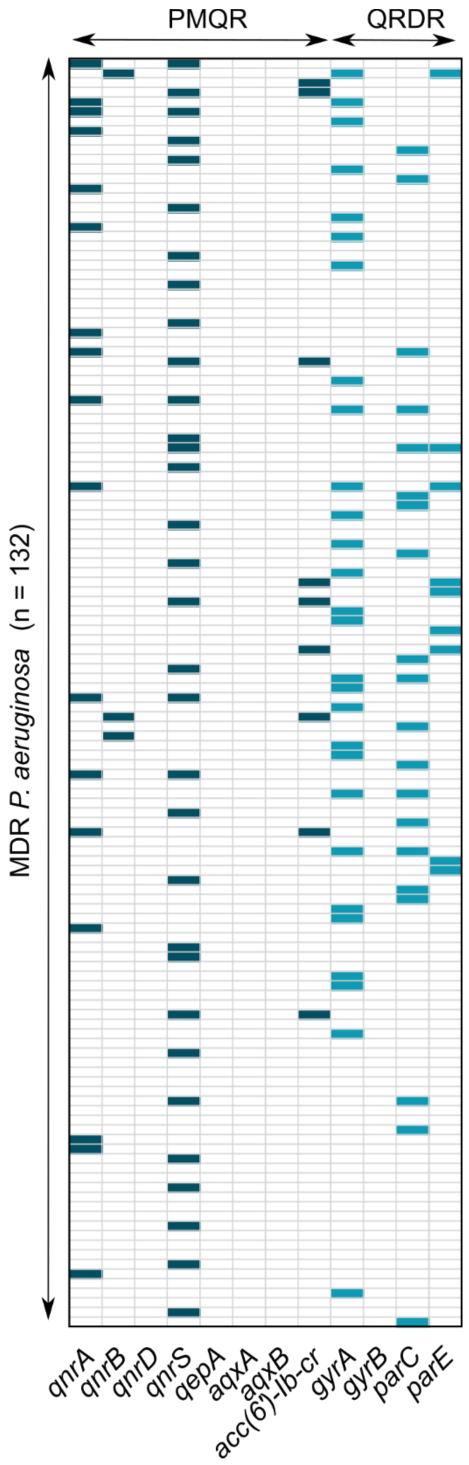

Supplementary Figure S1. Heatmap showing presence of PMQR (plasmid mediated quinolone resistance genes) and QRDR (chromosomal quinolone resistance determinants regions) in 132 multiple drug resistant clinical isolates of *Pseudomonas aeruginosa*.

Reference

37. CLSI. *Performance Standards for Antimicrobial Susceptibility Testing*, 13th ed.; CLSI Standard M02; Clinical and Laboratory Standards Institute: Wayne, PA, USA, 2018.
